# Dimensional accuracy, mechanical property, and optical stability of zirconia orthodontic bracket according to yttria proportions

**DOI:** 10.1038/s41598-023-47827-w

**Published:** 2023-11-21

**Authors:** Changbum Park, Hai-Van Giap, Jae-Sung Kwon, Kyung-Ho Kim, Sung-Hwan Choi, Joon Sang Lee, Kee-Joon Lee

**Affiliations:** 1 Department of Orthodontics, Institute of Craniofacial Deformity, Yonsei University College of Dentistry, No. 723, 50-1 Yonsei-ro, Seodaemun-gu, Seoul, 03722 Republic of Korea; 2 Department and Research Institute for Dental Biomaterials and Bioengineering, Yonsei University College of Dentistry, 50-1 Yonsei-ro, Seodaemun-gu, Seoul, 03722 Republic of Korea; 3 Department of Mechanical Engineering, Yonsei University, 50-1 Yonsei-ro, Seodaemun-gu, Seoul, 03722 Republic of Korea

**Keywords:** Dental materials, Orthodontics

## Abstract

This in vitro study evaluated comprehensively the performances of zirconia brackets with varying yttria proportions in manufacturing advanced orthodontic brackets. Three experimental groups of zirconia brackets were fabricated using yttria-stabilized zirconia (YSZ) materials with different yttria proportions—3 mol% yttria (3Y-YSZ), 4 mol% yttria (4Y-YSZ), and 5 mol% yttria (5Y-YSZ) (Tosoh Ceramic, Japan). A polycrystalline alumina ceramic bracket (3M™ Clarity™ Advanced, MBT 0.022-in. slot) was employed as the control group. Morphological properties, including slot surface structure and dimensions, were examined using scanning electron microscopy and surface profiler analysis. Manufacturing accuracy was assessed with root mean square calculations of trueness and precision. Mechanical properties were tested, encompassing static and kinetic frictional resistance (FR) and fracture strength. Optical stability was evaluated through 20,000 cycles of thermocycling and a 7-day immersion in various coloring agents. Within the limitations of this study, zirconia brackets containing 3 to 5 mol% YSZ presented enhanced reliability in terms of dimensional accuracy and demonstrated favorable optical stability. Notably, owing to its advantageous mechanical properties, the 3Y-YSZ variant showed remarkable potential as an advanced material for fabricating orthodontic brackets.

## Introduction

Ceramic brackets emerged in the 1980s as a viable solution for orthodontic appliances, elevating the aesthetic aspect of traditional stainless-steel brackets while upholding the treatment efficiency and favorable outcomes, especially in addressing complex malocclusion—the limitation of clear aligner therapy^1,2^. These brackets predominantly employ alumina, either in polycrystalline or monocrystalline form, depending on the manufacturing process. Despite offering aesthetic benefits, ceramic brackets present certain drawbacks, such as elevated friction resistance (FR) and diminished fracture toughness as compared to stainless-steel brackets^3,4^. These limitations often lead to challenges for orthodontists, including bracket wing fractures during clinical procedures. Such incidents can compromise enamel integrity and result in supplementary costs due to bracket replacement^4^. To address these limitations, various innovative approaches have been proposed to develop advanced orthodontic brackets, with zirconia emerging as a promising material for enhancing mechanical properties owing to its remarkable toughness^5,6^.

Zirconia brackets have been the subject of orthodontic research since the 1990s^7^. However, during this period, they did not demonstrate substantial improvements over alumina brackets in aspects such as frictional characteristics and aesthetic performance^8^. Recent advancements in zirconia materials have led to the development of several variants, influenced by factors such as powder selection, sintering additives, heat treatment, and other processing considerations^9^. Pure zirconia is composed of three main phases: monoclinic (m) at room temperature, tetragonal above 1170 °C, and cubic above 2370 °C^10^. While the monoclinic phase itself lacks remarkable mechanical attributes, the incorporation of dopants into the starting powder can augment strength and fracture toughness. This is achieved by partially stabilizing the tetragonal phase within the microstructure at ambient temperature^10^. Among the various dopants, yttria (Y_2_O_3_) stands out for its efficacy in providing a synergistic blend of robust strength and toughness, facilitating the stabilization of the tetragonal or cubic phase at room temperature^11,12^. Furthermore, yttria-stabilized zirconia (YSZ) allows for efficient production through computer-assisted design and fabrication (CAD/CAM) technologies. This ensures the reproduction of intricate details and lowered manufacturing costs, all while maintaining superior physical characteristics. As a result, 3 mol% yttria-stabilized tetragonal zirconia (3Y-YSZ) polycrystals have gained popularity in dental ceramics, particularly for prosthetic restorations. Recently, high-translucency partially stabilized zirconia with greater quantities of the non-birefringent cubic phase—achieved by utilizing higher yttria contents such as 4 mol% (4Y-YSZ) or 5 mol% (5Y-YSZ)—has been engineered. These innovations have notably broadened their clinical applications in terms of aesthetics^13^. Consequently, zirconia's emerging prominence in research and the creation of orthodontic brackets has become evident^5,6,14–16^. However, existing literature has reported a direct correlation between yttria concentration, translucency, and mechanical strength in zirconia restorations. While an increase in yttria concentration can stabilize the cubic phase, resulting in greater translucency, it may concurrently diminish the mechanical strength^17,18^. Therefore, to achieve a balance between mechanical properties and aesthetic demands in clinical applications, it becomes imperative to explore the performance and appropriateness of zirconia brackets with different yttria concentrations. To the best of our knowledge, the information available on this subject is scarce.

The objective of this in vitro study is to contribute to the existing knowledge base regarding the utilization of YSZ materials in the fabrication of advanced orthodontic brackets. The study involves a comprehensive examination and comparison of the morphological, mechanical, and optical attributes of zirconia brackets with those of commercial polycrystalline alumina brackets. The hypothesis was that there was no significant difference in the performance of zirconia brackets containing 3 to 5 mol% yttria proportions.

## Results

### Morphological characteristics

To quantify the slot dimensional measurement error, the Dahlberg error was computed^19^, confirming that the linear measurement error was 5.58 $\upmu$m, while the angulation measurements ranged from 0.10° to 0.27°.

Each group's bracket dimensions were measured and presented in Table 1. No disparities were observed in any dimensional parameters between the experimental groups; however, significant differences emerged between the control and experimental groups. The experimental groups manifested a greater slot base width (mean difference, 31.2 to 34.1 $\upmu$m) and a reduced slot base angle (mean difference, 1.83° to 2.42°). Furthermore, although essentially parallel slot walls were verified in all groups, the experimental groups exhibited a lesser divergence of slot walls, attributable to an approximately 1° inward tilt of the upper angle relative to the control group.

**Table 1:** Slot dimensions of the experimental and control groups.

Parameters	Control group	Experimental groups				Sig
		3Y-YSZ	4Y-YSZ	5Y-YSZ	p	
Slot base width	420.40 $\pm$ 4.05	453.60 $\pm$ 3.25	454.50 $\pm$ 3.26	452.70 $\pm$ 3.97	0.408	0.000*
LA	74.12 $\pm$ 0.56	74.51 $\pm$ 0.53	74.61 $\pm$ 0.59	74.48 $\pm$ 0.43	0.850	0.194
UA	75.16 $\pm$ 0.62	73.77 $\pm$ 0.68	73.95 $\pm$ 0.58	73.75 $\pm$ 0.57	0.744	0.000*
SBA	20.39 $\pm$ 0.57	18.35 $\pm$ 0.75	18.35 $\pm$ 0.61	17.97 $\pm$ 0.58	0.325	0.000*

Data are shown as mean $\pm$ standard deviation (linear: $\upmu$m, angular: °).

UA, upper angle; LA, lower angle; SBA, slot angle.

Intergroup comparisons were performed using one-way ANOVA.

*Statistically significant at p < 0.01.

The trueness and precision of all morphological parameters were assessed across all groups, with the findings summarized in Tables 2 and 3.

**Table 2:** RMS trueness values in the experimental and control groups.

	Parameters	Group	Mean $\pm$ SD	p	Sig
Trueness	Slot base width	Control	61.6 $\pm$ 4.05	0.000**	0.000*
		3Y-YSZ	117.40 $\pm$ 3.25	0.000**	
		4Y-YSZ	115.50 $\pm$ 3.26	0.000**	
		5Y-YSZ	117.30 $\pm$ 3.78	0.000**	
	SBA	Control	3.39 $\pm$ 0.57	0.000**	0.000*
		3Y-YSZ	0.57 $\pm$ 0.59	0.170	
		4Y-YSZ	0.59 $\pm$ 0.34	0.097	
		5Y-YSZ	0.50 $\pm$ 0.23	0.870	

Data are shown as mean $\pm$ standard deviation of root means square values calculated for trueness (linear: $\upmu$m; angular: °).

SBA, the slot angle.

A one-sample T-test was performed to assess the trueness.

Intergroup comparisons were performed using one-way ANOVA.

*Statistically significant at p < 0.01.

**Table 3:** RMS values of precision in the experimental and control groups.

	Parameters	Group	Mean $\pm$ SD	Sig.
Precision	Slot base width	Control	4.82 $\pm$ 3.13	0.200
		3Y-YSZ	3.84 $\pm$ 2.54	
		4Y-YSZ	3.83 $\pm$ 2.62	
		5Y-YSZ	4.69 $\pm$ 3.13	
	UA	Control	0.72 $\pm$ 0.50	0.891
		3Y-YSZ	0.76 $\pm$ 0.59	
		4Y-YSZ	0.68 $\pm$ 0.47	
		5Y-YSZ	0.69 $\pm$ 0.44	
	LA	Control	0.63 $\pm$ 0.48	0.191
		3Y-YSZ	0.64 $\pm$ 0.41	
		4Y-YSZ	0.69 $\pm$ 0.48	
		5Y-YSZ	0.50 $\pm$ 0.36	
	SBA	Control	0.65 $\pm$ 0.49	0.202
		3Y-YSZ	0.87 $\pm$ 0.63	
		4Y-YSZ	0.69 $\pm$ 0.51	
		5Y-YSZ	0.68 $\pm$ 0.46	

Data are shown as mean $\pm$ standard deviation of root means square values calculated for precision (linear: $\upmu$m; angular: °).

UA, upper angle; LA, lower angle; SBA, slot angle.

Intergroup comparisons were performed using one-way ANOVA.

*Statistically significant at p < 0.01.

In the control group, the bracket slot width exceeded the nominal values defined by the manufacturers by approximately 61.6 $\upmu$m (11.02%; p < 0.05), and the slot base angle was larger by approximately 3.39° (slot width, 558.8 $\upmu$m; slot base angle, 17°; p < 0.05). Conversely, in the experimental groups, the bracket slot width was less than their *digital reference* design's value (slot width, 770 $\upmu$m). The mean disparities for the 3Y-YSZ, 4Y-YSZ, and 5Y-YSZ groups were 117.40 $\upmu$m (15.25%), 115.50 $\upmu$m (15.00%), and 117.30 $\upmu$m (15.26%), respectively (p < 0.05). These measurements underscore the linear shrinkage of the YSZ material during sintering, a phenomenon anticipated through detailed observation to regulate the experimental groups' slot dimensions. As a result, a higher trueness value for the slot base width was corroborated in the experimental group (p < 0.05). However, the slot base angle in the experimental groups did not differ significantly from the reference design (slot base angle, 18°) (Table 1), resulting in a lower trueness value in the experimental groups relative to the control group (p < 0.05) (Table 2). Moreover, the precision of any morphological parameter did not vary among the groups (Table 3).

**Figure 1:**
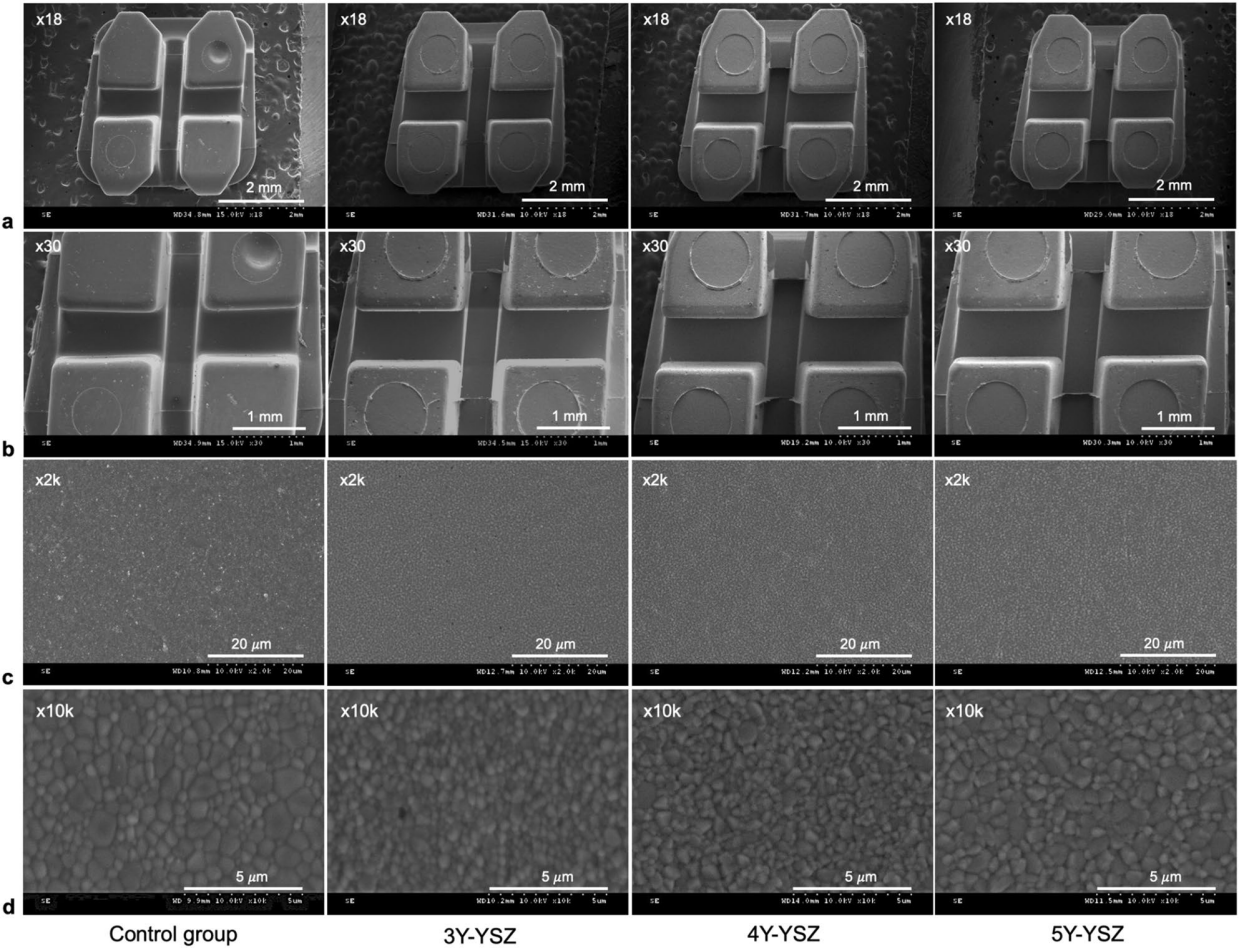
Comprehensive representation of the experimental and control group structures at various magnifications. (**A**) ×18, (**B**) ×30, (**C**) ×2k, and (**D**) ×10k.

Figure 1 illustrates the general structures of the bracket groups. The bracket slot surfaces displayed no apparent defects and were characterized by a smooth polycrystalline surface with uniform grains. The mean average grain size increased with the increase of yttria content. No significant difference in the average grain size was observed between the control, 4Y-YSZ, and 5Y-YSZ groups (Table 4).

**Table 4:** The average grain size in the experimental and control groups.

Group	Grain size	p
Control	0.639 $\pm$ 0.073^a^	0.000*
3Y-YSZ	0.374 $\pm$ 0.063^b^	
4Y-YSZ	0.580 $\pm$ 0.021^a^	
5Y-YSZ	0.682 $\pm$ 0.102^a^	

Data are shown as mean $\pm$ standard deviation (nm).

Intergroup comparisons were performed using one-way ANOVA.

The same superscript letters indicate no significant differences between groups (Tukey’s multiple comparison test).

*Statistically significant at p < 0.01.

The surface roughness of the bracket slot was subsequently assessed using surface profiler analysis, affirming the lowest surface roughness parameters for 3Y-YSZ (Ra = 45.61; Rq = 56.76; p < 0.05). No marked differences in surface roughness were identified between the 3Y-YSZ and 4Y-YSZ groups or the control and 5Y-YSZ groups (Table 5).

**Table 5:** Surface roughness values of experimental and control groups.

Group	Ra	p	Rq	p
Control	57.02 $\pm$ 7.44^a^	0.000*	71.57 $\pm$ 8.67^c^	0.000*
3Y-YSZ	45.63 $\pm$ 6.61^b^		56.76 $\pm$ 8.76^d^	
4Y-YSZ	50.87 $\pm$ 5.39^a,b^		64.70 $\pm$ 7.68^c,d^	
5Y-YSZ	60.72 $\pm$ 5.63^a^		76.44 $\pm$ 7.08^c^	

Data are shown as mean $\pm$ standard deviation ($\upmu$m).

Ra, roughness average, Rq, root mean square.

Intergroup comparisons were performed using one-way ANOVA.

The same superscript letters indicate no significant differences between groups (Tukey’s multiple comparison test).

*Statistically significant at p < 0.01.

### Mechanical properties

**Figure 2:**
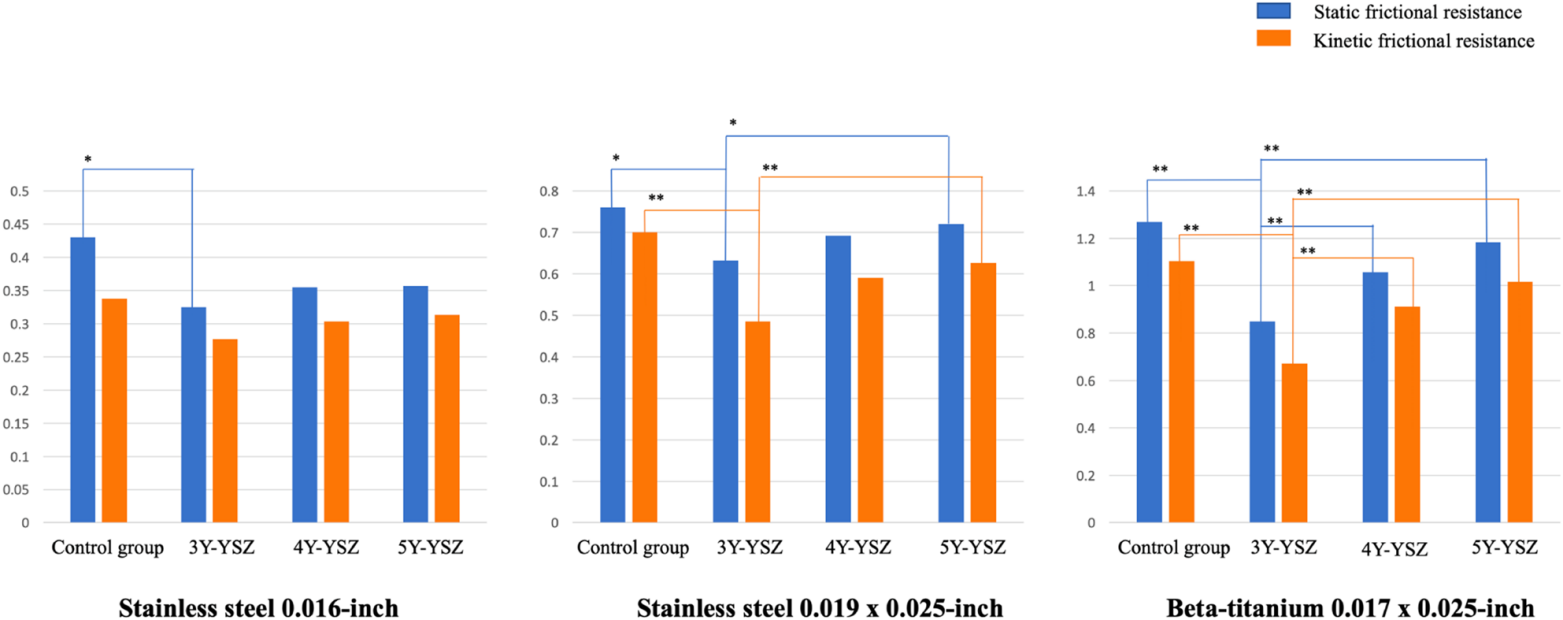
Graphical display of static and kinetic friction values for both the experimental and control groups.

The static and kinetic FR values varied significantly among the groups, displaying a consistent pattern. Within the groups, the 3Y-YSZ group demonstrated the least frictional forces for all wire types tested. The control group recorded the highest friction forces, although no considerable differences were found between the control, 4Y-YSZ, and 5Y-YSZ groups (Fig. 2).

The fracture strength of bracket tie wings was examined in each bracket group through tensile tests. Among them, the 3Y-YSZ group presented the highest fracture strength of the bracket tie wing, followed by the control group (p < 0.05). No significant difference in the fracture strength of the bracket tie wing was observed between the 4Y-YSZ and 5Y-YSZ groups (Table 6).

**Table 6:** Fracture strength of bracket tie wings in the experimental and control groups.

Group	Fracture strength	95% Confidence Interval for Mean		p
		Lower Bound	Upper Bound	
Control	132.68 ± 7.13^a^	127.58	137.77	0.000*
3Y-YSZ	141.51 ± 5.30^b^	137.72	145.30	
4Y-YSZ	75.3 ± 7.36^c^	70.05	80.58	
5Y-YSZ	67.18 ± 8.29^c^	61.24	73.11	

Data are presented as mean $\pm$ standard deviation (MPa).

Upper superscript letters indicate intergroup comparisons (One-way ANOVA). The same upper superscript letters indicate no significant differences between groups (Tukey’s multiple comparison test).

*Statistically significant at p < 0.01.

### Optical properties and influence of aging on optical stability

Prior to testing, the control group demonstrated the highest direct light transmission, succeeded by the 5Y-YSZ group. There was no significant variation in percentage transmittance between the 3Y-YSZ and 4Y-YSZ groups. Post-testing, all groups exhibited a reduction in transmittance percentage, with the 5Y-YSZ group manifesting pronounced alterations following a 7-day immersion in tea, coffee, and red wine solutions (Table 7).

**Table 7:** Percentage of transmittance of experimental and control groups over time.

t%	Control	3Y-YSZ	4Y-YSZ	5Y-YSZ	P^§^
Before thermocycling	64.35 ± 5.99	30.26 ± 1.27^a^	29.34 ± 2.27^a^	44.74 ± 1.91_A_	0.000*
After thermocycling	64.27 ± 2.18	31.69 ± 0.83^b^	30.57 ± 1.98^b^	43.49 ± 2.52	0.000*
Coke	59.24 ± 1.73	30.99 ± 0.60^c^	30.10 ± 1.12^c^	42.29 ± 2.52	0.000*
Wine	60.31 ± 1.87	26.86 ± 0.83^d^	29.17 ± 1.91^d^	40.74 ± 1.34_B_	0.000*
Coffee	56.54 ± 2.03	28.59 ± 0.79^e^	27.85 ± 1.41^e^	39.20 ± 1.15_C_	0.000*
Tea	59.43 ± 3.26	27.75 ± 1.34^f^	24.82 ± 0.98^f^	39.35 ± 1.89_D_	0.000*
Sig. ^†^	N.S	N.S	N.S	A > B, C, D	

Data are shown as mean $\pm$ standard deviation.

^§^Intergroup comparisons were tested by One-way ANOVA. The same upper superscript letters indicate no significant differences between groups (Tukey’s multiple comparison test).

^†^Changes in the transmittance percentage before thermocycling and after each testing solution were compared by pair T-test in each group.

*Statistically significant at p < 0.01.

N.S. = not significant.

Significant color changes occurred in all bracket groups, reaching up to 2.75 units in the control group and 3.46 units in the experimental groups, predominantly in coffee and tea solutions. However, no marked intergroup differences in the degree of color change (${\Delta E}_{ab}^{*}$) were detected under the experimental conditions (Table 8, Fig. 4).

**Table 8:** Changes in reflected color (${\Delta E}_{ab}^{*}$) and color parameters of the experimental and control groups.

Group	∆E*_ab_	p	∆L*	p	∆a*	p	∆b*	p
Thermal cycling								
Control	0.39 ± 0.14	0.794	− 0.15 ± 0.36	0.737	− 0.05 ± 0.06	0.962	0.11 ± 0.19	0. 558
3Y-YSZ	0.54 ± 0.25		− 0.14 ± 0.61		− 0.04 ± 0.06		0.12 ± 0.11	
4Y-YSZ	0.50 ± 0.37		− 0.34 ± 0.49		− 0.03 ± 0.06		0.19 ± 0.13	
5Y-YSZ	0.53 ± 0.21		− 0.39 ± 0.18		− 0.03 ± 0.06		0.27 ± 0.29	
Coke								
Control	1.25 ± 0.75	0.246	0.93 ± 1.03	0.060	− 0.02 ± 0.09	0.002*	0.49 ± 0.25	0.095
3Y-YSZ	0.92 ± 0.30		− 0.24 ± 0.83		0.16 ± 0.06		0.53 ± 0.04	
4Y-YSZ	1.08 ± 0.43		− 0.25 ± 0.83		0.11 ± 0.04		0.80 ± 0.25	
5Y-YSZ	0.62 ± 0.33		− 0.40 ± 0.27		0.10 ± 0.04		0.41 ± 0.31	
Wine								
Control	1.15 ± 0.51	0.126	− 0.86 ± 0.78	0.202	0.12 ± 0.07	0.000*	0.50 ± 0.21	0.588
3Y-YSZ	1.22 ± 0.28		− 0.88 ± 0.30		0.40 ± 0.08		0.72 ± 0.13	
4Y-YSZ	1.65 ± 0.66		− 1.31 ± 0.77		0.50 ± 0.13		0.70 ± 0.41	
5Y-YSZ	1.75 ± 0.23		− 1.55 ± 0.20		0.50 ± 0.05		0.62 ± 0.25	
Coffee								
Control	2.22 ± 0.56	0.214	− 1.11 ± 0.91	0.225	0.11 ± 0.07	0.000*	1.80 ± 0.17	0.089
3Y-YSZ	2.64 ± 0.74		− 1.65 ± 0.90		0.35 ± 0.08		1.95 ± 0.28	
4Y-YSZ	2.32 ± 0.45		− 1.62 ± 0.58		0.43 ± 0.14		1.55 ± 0.20	
5Y-YSZ	2.96 ± 0.55		− 2.12 ± 0.38		0.48 ± 0.08		2.01 ± 0.42	
Tea								
Control	2.75 ± 0.56	0.319	− 1.84 ± 0.70	0.289	0.27 ± 0.05	0.000*	1.99 ± 0.18	0.549
3Y-YSZ	3.27 ± 0.71		− 2.55 ± 0.86		0.62 ± 0.08		1.88 ± 0.18	
4Y-YSZ	3.36 ± 0.79		− 2.42 ± 0.98		0.73 ± 0.14		2.14 ± 0.21	
5Y-YSZ	3.46 ± 0.38		− 2.74 ± 0.22		0.66 ± 0.07		1.98 ± 0.44	

Data are shown as mean $\pm$ standard deviation.

Intergroup comparisons were performed using one-way ANOVA.

*Statistically significant at p < 0.01.

## Discussion

Recent advancements in the composition, structure, and fabrication techniques of zirconia material have substantially enhanced its mechanical properties and aesthetic features, particularly within dental prosthodontics^9^. This evolution has fueled an increasing interest in employing zirconia in the manufacture of orthodontic brackets, surpassing the constraints of conventional alumina ceramic brackets^5,6,14–16^. However, the performance of orthodontic appliances fabricated by these novel zirconia variants remained unclear. Our study serves as a pioneering effort to bridge this gap by offering crucial insights into the performance of zirconia brackets. The study spans an exploration of varying yttria proportions and their implications for developing advanced orthodontic brackets. The findings emphasize that zirconia brackets, containing 3 to 5 mol% YSZ, demonstrate superior reliability in dimensional accuracy compared to the control group. Additionally, our analysis reveals significant variations in the mechanical and optical properties of the brackets, depending on the yttria proportions. The null hypothesis was therefore rejected.

To mitigate confounding factors in the experimental results, the zirconia brackets utilized in this study were digitally designed using the reverse engineering process, adhering to the morphology of the control group. Despite the absence of any significant disparity in slot dimension among the zirconia groups, they exhibited a more substantial slot base width (mean difference, 31.2 to 34.1 $\upmu$m), a diminished slot base angle (mean difference, 1.83° to 2.42°), and reduced divergence of slot walls relative to the control group (Table 1). These variations can be attributed to slight discrepancies in the digital design of the zirconia brackets when compared with the morphology of the control group. To a certain extent, such differences appear unavoidable because of errors encountered during the fabrication processes (Fig. 4).

In this study, the precision and trueness of the fabrication process were assessed by calculating the RMS values. According to these trueness measurements, the control group's dimensions were larger by approximately 61.6 $\upmu$m in slot width (11.02%) and approximately 3.39° in slot base angle compared to the nominal values, with a significance level of p < 0.05 (Table 2). These findings align with previous studies^20^. For instance, Lefebvre et al.^21^ examined the accuracy of several commercial brackets and concluded that over 90% of slot width measurements deviated by up to 24% from the values stated by the manufacturers, along with inconsistent slot inclination angles. Within the field of orthodontics, achieving precise slot dimensions is critical, as it directly influences the effectiveness of the torque exerted on the teeth. To create an efficient pre-adjusted bracket and reduce compensatory bending, manufacturers must focus on precision, especially with regard to slot dimensions^22^. Of all ceramic materials, zirconia is known for its exceptional fracture toughness, rendering it suitable for meticulous shaping and thereby potentially enhancing accuracy and reproducibility in finer details^1,5,8^. In fact, the trueness values for the slot base angle in the experimental groups showed no significant deviation from the *digital reference design* values (Table 2), reflecting the higher level of accuracy attained during the manufacturing process across all yttria proportions. Furthermore, the precision values for all slot dimension parameters in the experimental groups were found to be consistent and reproducible. Adhering to the ISO 27020 standard (ISO 27020, 2010), with tolerances of $\pm$ 0.01 mm in slot width, $\pm$ 1° in torque, and $\pm$ 1° in slot wall parallelism^23^, the slot dimensional accuracy in the experimental groups was confirmed with elevated reliability (Tables 2 and 3).

In the field of orthodontics, it is crucial to comprehend the force necessary to overcome friction at the bracket–archwire interface, as this understanding aids in producing optimal biological tooth movement^3^. Our findings indicated that both FR values and the surface roughness of the zirconia brackets are influenced by the yttria proportion; specifically, the 3Y-YSZ group exhibited the smoothest surface and the lowest static and kinetic friction values under all tested conditions (Tables 5 and Fig. 2)^24–26^. These variations among the zirconia bracket groups became particularly pronounced when larger sizes of rectangular archwires were employed or when archwires made of alloys with rougher surfaces, such as TMA archwires, were utilized^3,27^. Compared to the control group, zirconia brackets demonstrated reduced FR values; however, the significant differences were solely observed with the 3Y-YSZ group (Fig. 2).

Fracture strength is another vital mechanical property that holds relevance to the clinical functionality of ceramic brackets. The manufacturing process is instrumental in defining the ceramics' strength; hence, it is advised to test actual ceramic brackets rather than bulk bracket materials^4^. All bracket groups in the present study were true-twin brackets, fabricated through the injection molding process, and maintained uniformity in size and shape to regulate the factors that could influence the brackets' fracture strength^4^. The 3Y-YSZ group presented statistically the highest mean maximum tie-wing fracture strength (Table 6). This outcome can be attributed to the distinctive transformation toughening characteristic of zirconia material, where stress induces a phase shift from the tetragonal to monoclinic phase at the crack tip. This transformation, accompanied by a resultant increase in volume, modifies crack propagation and thereby augments the material's fracture resistance^6,28^. However, zirconia stabilized with an elevated yttria content leads to an increase in cubic content, thus resulting in reduced fracture toughness^6,17,18,29^. Unlike zirconia, alumina brackets lack these protective mechanisms^6^. Consequently, the utilization of 3Y-YSZ brackets in orthodontic treatment might prove advantageous because of the diminished incidence of wing fracture.

**Figure 3:**
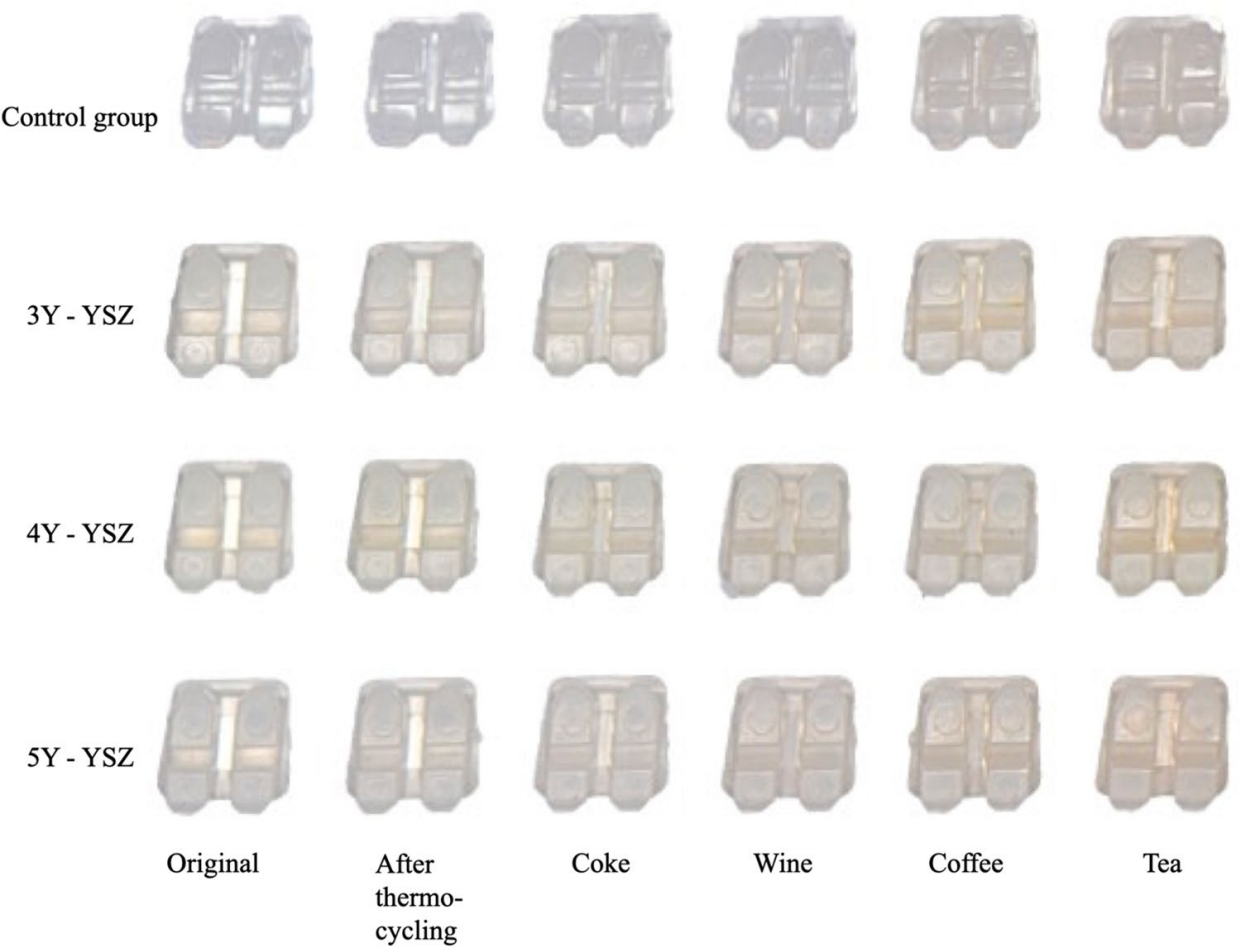
Changes in the appearance of the experimental and control bracket groups over time.

The aesthetic appeal of orthodontic fixed appliances has long been a concern for patients. To get a good aesthetic appearance, the bracket should match the underlying tooth color and/or possess high translucency^30^. In the current study, the 5Y-YSZ group displayed a significantly enhanced translucency compared to the 3Y-YSZ and 4Y-YSZ groups, yet all experimental groups were less translucent than the control group (Table 7)^31^. Therefore, to achieve a visually imperceptible appearance, options such as coloring the brackets to achieve desirable color-matching with patients' teeth and/or enhancing the translucency of the zirconia bracket can be considered. Nevertheless, to maintain an aesthetic appearance, good optical stability is required^30,32^. In this study, changes in transmittance percentage were detected across all groups, with substantial differences noted in the 5Y-YSZ group following a 7-day immersion in tea, coffee, and red wine solutions (Table 7). Likewise, significant alterations in the coloration of all bracket groups were observed, but without noticeable differences between the groups (Table 8). These findings indicate that the yttria proportion influences the translucency of the YSZ material, but not its resistance to staining or discoloration^32,33^. Even though substantial variations in optical properties were registered, acceptable color stability was confirmed in both control and experimental groups according to a threshold of 3.7 ${\Delta E}_{ab}^{*}$ units for clinically perceptible color change (Fig. 3).

This study revealed that the fabrication process of YSZ brackets consistently demonstrated high accuracy and reproducibility across various yttria proportions, underscoring their potential for achieving predictable orthodontic tooth movement. Moreover, the YSZ material holds promise for fabricating self-ligating brackets, a category that places particular emphasis on intricate manufacturing details. Among the zirconia bracket categories, the 3Y-YSZ brackets stood out for their superior mechanical performance, marked by reduced FR forces and enhanced surface integrity in response to fracture loads. Such attributes offer advantages in optimizing orthodontic treatment. While the color stability of zirconia brackets was verified and the translucency of YSZ significantly augmented by raising the yttria proportion in the 5Y-YSZ bracket, further advancements in translucency or color-matching strategies may be required to satisfy patient preferences without compromising the exceptional physical and mechanical characteristics of the zirconia material^15,34^. The combination of different YSZ materials, such as composing the 3Y-YSZ and 5Y-YSZ, can be an option to achieve an esthetic appearance while optimizing exceptional physical and mechanical characteristics^35,36^.

This in vitro study has some limitations. Firstly, it must be noted that the results obtained in this study may not wholly represent the clinical scenario, owing to limitations inherent in laboratory conditions, despite measures taken to control for confounding variables^30^. Additional clinical investigations are warranted to investigate the effects of these material properties in daily orthodontic practice. Secondly, the bonding strength of zirconia brackets was not included within the scope of this study since it is not only related to the material itself but also the design of the bracket base, surface treatment, and adhesive materials^37,38^. Therefore, the bonding strength of these novel zirconia brackets and the enamel surface integrity after debonding should be investigated in the future.

## Methods

### Design and manufacture of zirconia brackets

The experimental groups were fabricated utilizing three distinct zirconia powders: 3Y-YSZ (Zpex; Tosoh Ceramic, Japan), 4Y-YSZ (Zpex4; Tosoh Ceramic, Japan), and 5Y-YSZ (ZpexSmile; Tosoh Ceramic, Japan). According to the manufacturing specifications, all these powders are highly translucent zirconia grades, containing less than 0.1 wt% alumina, with the primary distinction being the yttria content^39^.

**Figure 4:**
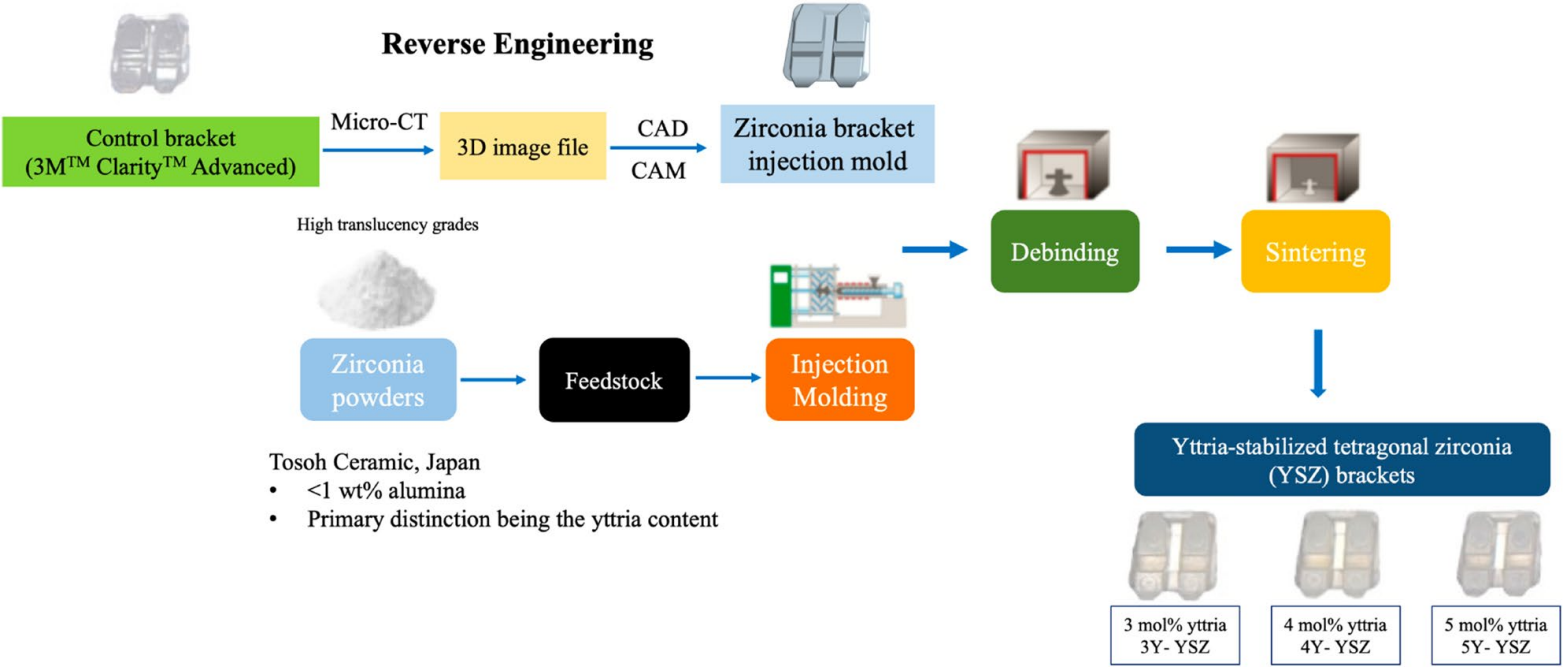
Schematic diagram illustrating the ceramic injection molding method utilized for the production of zirconia specimens.

All brackets were meticulously fabricated for the maxillary right central incisors, employing the reverse engineering process based on the morphology of an existing polycrystalline alumina ceramic bracket product (3M™ Clarity™ Advanced, MBT 0.022-inch slot) used as the control group. Figure 4 delineates the ceramic injection molding method employed for the production of the zirconia brackets. During this procedure, the mold was digitally conceptualized through three-dimensional (3D) software (Creo 5.0, PTC, USA)—referred to as *the digital reference design*—based on a 3D scanned image of the control group's morphology (micro-computed tomography scanner, SkyScan 1173, Bruker, USA). This design process took into consideration the linear shrinkage associated with zirconia materials.

## Measurements

### Morphological properties

#### Scanning electron microscopy (SEM) analysis

For the SEM analysis, samples of 10 brackets were randomly selected from each group and studied using an SEM instrument (S-3000N, Hitachi, Tokyo, Japan). The focus of this analysis was to examine the dimensions and surface structure of the bracket slots. The specimens were secured on SEM stubs, subjected to drying in a freeze dryer (ES-2030, Hitachi, Tokyo, Japan), and then coated with platinum to a thickness of 100 nm using an ion coater (E-1010, Hitachi, Tokyo, Japan). Photomicrographs were captured from each bracket's face at an operating voltage of 15 kV. Low-magnification SEM images provided insights into the overall structure, while high-magnification SEM images revealed detailed microcosmic surface topography^40^. The average grain size was investigated by the line intercepted method at the magnification of 10k in which five micrographs and five lines were used for each bracket group^41,42^.

**Figure 5:**
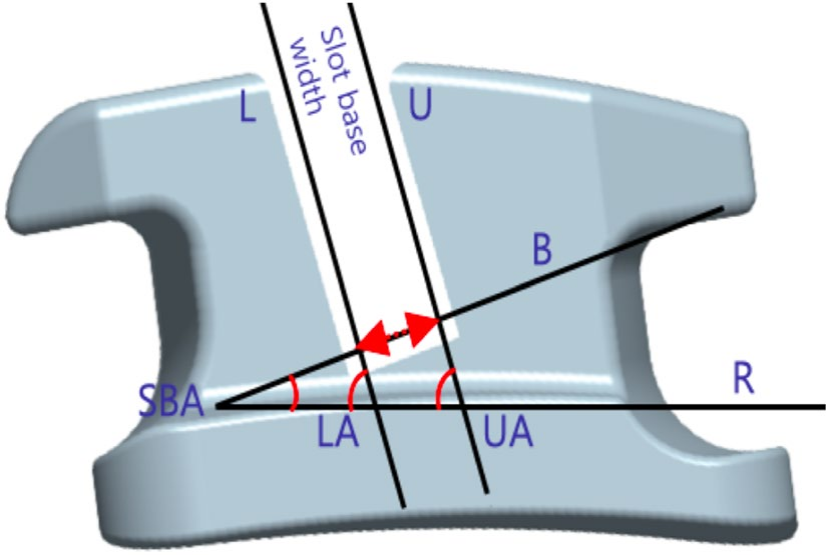
Detailed illustration of bracket slot measurements. R represents a horizontal reference line; B is a line parallel to the slot base, distanced 100 μm from it; U is a line parallel to the upper wall of the slot, positioned 100 μm from that wall; L is a line parallel to the lower wall of the slot, distanced 100 μm from it; slot angle (SBA) denotes the angle between R and B; upper angle (UA) signifies the angle between R and U; lower angle (LA) characterizes the angle between R and L.

The slot dimensions in each bracket group were assessed in lateral views using a computer-based measuring tool (IMT i-Solution Inc., version 7.3; Coquitlam, BC, Canada). To mitigate any bias from the rounded nature of the slot angles, measurements were carried out at a distance of 100 μm from the base and wall of the slot. Subsequent measurements included the slot base width, slot angle (corresponding to the torque of the bracket prescription), and parallelism of the slot walls (Fig. 5)^40^.

The precision and trueness of the slot dimensions were evaluated to ascertain the dimensional accuracy of the brackets. Precision, defined as the agreement of repeated results, was calculated by comparing the differences among pairs within the 10 brackets of each group. Trueness, indicating the agreement of the slot dimension with a true value, was determined by contrasting the dimensions of the control group's 10 brackets with the manufacturers' specified nominal values, while in the experimental group, the dimensions were compared with the *digital reference design*. To compensate for the offset error due to positive and negative value deviations, root mean square (RMS) values were computed for both precision and trueness^43^. A lower RMS value for precision or trueness is indicative of higher accuracy.

### Surface profiler analysis

The examination of surface roughness in the bracket slots was conducted using a surface profiler (DektakXT Stylus Profiler, Bruker, USA), with a sample of 10 brackets per group. Each bracket was sectioned using a fine diamond disk. The profiler was operated with an inductive gauge that featured a 12.5-$\upmu$m-radius diamond stylus, moving at a scanning speed of 5 m/s. Prior to the examination, all brackets were meticulously cleaned with 95% alcohol. The specimens were scanned to evaluate two key surface roughness parameters: the average roughness (Ra) and RMS roughness (Rq).

### Mechanical properties

#### Friction resistance (FR) tests

A designated sample comprising 30 bracket–wire combinations was prepared for each group (refer to Table 9), wherein an elastic ligature (Ormco) was utilized to secure the archwire to the bracket, applied consistently by the same individual. To negate the effect of ligature force decay, the elastomeric rings were affixed immediately preceding each test. Both bracket and archwire specimens were meticulously cleaned with 95% alcohol prior to examination.

**Table 9:** Design of the frictional resistance test.

Wire alloys	Wire sections	Control group	Experimental groups		
			3Y-YSZ	4Y-YSZ	5Y-YSZ
Stainless steel	0.016-in.	10	10	10	10
	0.019 in. × 0.025-in.	10	10	10	10
Beta-titanium	0.017 × 0.025-in.	10	10	10	10

The investigation of the FR was conducted in a dry state using a universal testing machine (Instron 5942; Instron Corp., USA)^44^. The bracket slot and wire were positioned at an angulation of 0°, and the wire was drawn through the slot for a distance of 5 mm at a crosshead speed of 5 mm/min. The resulting static and kinetic friction forces were recorded. Specifically, the static frictional force was ascertained from the initial force peak, while the kinetic frictional force was computed as the average force subsequent to the peak until the conclusion of the test.

### Fracture strength of the bracket tie wings

**Figure 6:**
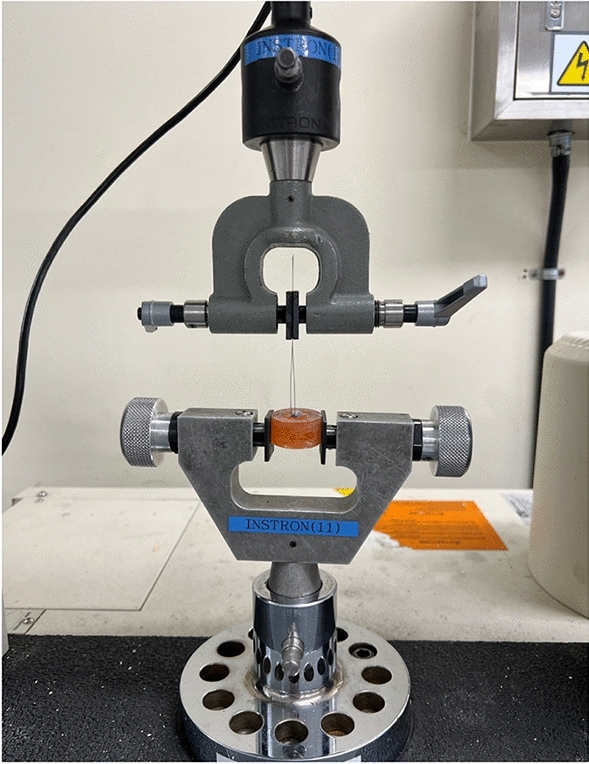
Mechanical testing apparatus.

**Figure 7:**
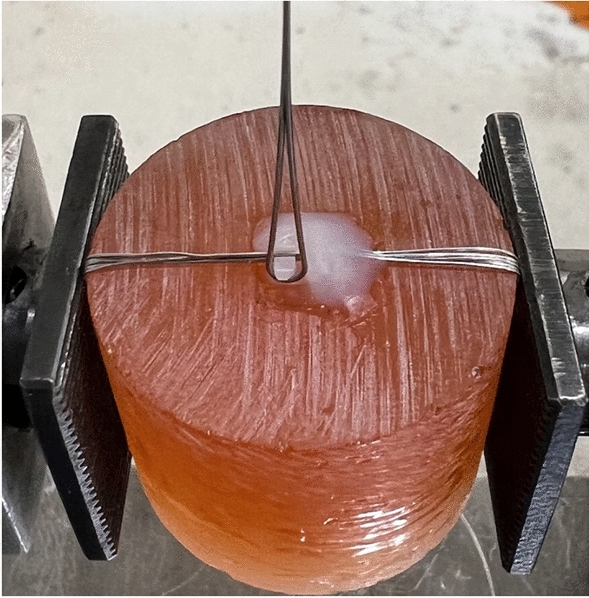
The bracket was bonded to an acrylic mold. TransbondTM XT Light Cure Adhesive Primer (3M) was applied to both the acrylic mold and bracket base using a brush. A composite resin (TransbondTM XT Light Cure Adhesive Paste—3M) was used to bond brackets to acrylic molds, followed by 20 s of light polymerized. To enhance the stabilization, 0.010-inch steel ligature tie wire (TP Orthodontics, Inc., USA) was tied around, then the composite resin was added over the bracket surface and flowed onto the acrylic mold so that no composite resin flowed under the tested tie wing. The composite resin was light polymerized from 5 directions (mesial, distal, gingival, incisal, and facial) for 40 s each.

Each group's sample of ten brackets was subjected to a fracture strength test. Brackets were bonded to acrylic molds which were fixed firmly with the lower tensile grip within a universal testing machine (Instron 3366; Instron Corp., USA). A 0.016-inch stainless steel wire (Ormco) was looped under the distoincisal tie wing and affixed to the upper tensile grip (Figs. 6 and 7).

The distoincisal tie wing was tested to failure at a crosshead speed of 10 mm/min. The fracture strength of bracket tie wings (MPa) was calculated by dividing the tensile load at failure (N) by the area of contact between the wire and tie wing (mm^2^)^4^. The area of contact was calculated by using a 3D Inspection Microscope (Hirox KH-1000 Inc., USA) at a magnification of 50×.

### Optical properties and optical stability

The color and translucency properties of the brackets were scrutinized using both reflection and transmission methodologies with a spectrophotometer (CM-5, Konica Minolta, Japan), employing a D65 light source. Calibration of the spectrophotometer was diligently executed according to the manufacturer's directives before initiating the measurements.

The stability of the optical characteristics of the brackets was examined through a process of artificial aging and exposure to coloring substances found in commonplace beverages. Artificial aging was implemented through 20,000 cycles of thermocycling, each with a 30s-dwell time in distilled water at temperatures of 5 °C and 55 °C. This process aimed to emulate 2 years of clinical utilization within the oral cavity, in accordance with the recognized average duration of orthodontic treatment^45^. Following this, the brackets were immersed for a 7-day period in various staining solutions, including coffee (Maxim Arabica 100, South Korea), red wine (Bourgogne Hautes-Côtes de Nuits Les Dames de Vergy 2018, France), coke (Coca-Cola Zero, South Korea), and black tea (Starbucks Teavana Earl Grey Black Tea, South Korea)^46^.

Baseline color and translucency were documented to investigate alterations following thermocycling and immersion in coloring agents. Measurements were obtained from randomly selected brackets (10 from each group), subsequent to cleansing with distilled water to remove any residual dye waste.

Direct transmission analysis was conducted three times within the 400–700 nm wavelength range (visible light spectrum). Each specimen was shielded with an opaque black cardboard mask, featuring a central window for measurement, and the mean value was subsequently determined^30^.

Reflection analysis adhered to the Commission Internationale de l’Eclairage (CIE) L* a* b* (LAB) color scale, where L* represents brightness (from black to white), a* denotes the color value from green to red, and b* signifies the color value from yellow to blue. Five assessments were recorded with a measuring aperture diameter of 3 mm at the labial surface center of the bracket and averaged to ascertain the value for each specimen. To prevent the influence of variation in the background, the zero-calibration box was used to block external light during measurements. Color alterations before and after testing were computed using the Eq. (1)^32,47^:

1 $${\Delta E}_{ab}^{*}=[{{{(\Delta L}^{*})}^{2}+{{(\Delta a}^{*})}^{2}+{{(\Delta b}^{*})}^{2}]}^\frac{1}{2}.$$

### Statistical analysis

The measurement repeatability and intra-observer variability were evaluated by computing the intraclass correlation coefficient between two assessments taken at 2-week intervals by a single inspector. The ensuing intraclass correlation coefficient (ICC) between the measurement pairs denoted high reliability (ICC > 0.97).

To verify the data distribution's normality, the Shapiro–Wilk test was administered. A one-sample T-test analysis was utilized to examine intragroup disparities in trueness. One-way ANOVA and post-hoc Tukey’s multiple comparison tests were employed to analyze the intergroup variations in morphological, mechanical, and optical parameters. All statistical evaluations were conducted with SPSS 24.0 Statistical Software (SPSS, Armonk, NY, USA), applying a significance threshold of 0.05.

## Data Availability

The datasets used and/or analyzed during the current study are available from the corresponding author upon reasonable request.
